# The synergy between diurnal temperature range and calcium concentration help to predict hospital mortality in patients with acute myocardial infarction

**DOI:** 10.1038/s41598-022-18816-2

**Published:** 2022-09-15

**Authors:** Xingbo Gu, Dandan Liu, Ning Hao, Xinyong Sun, Shulei Liu, Xiaoxu Duan, Shuang Yang, Jia Li, Shu Wang

**Affiliations:** 1 Department of Cardiology, the First Affiliated Hospital, Cardiovascular Institute, Harbin Medical University, Harbin, 150001 People’s Republic of China; 2 Department of Biostatistics, International School of Public Health and One Health, Hainan Medical University, Haikou, 571199 People’s Republic of China; 3 Department of Cardiology, the 210th Hospital of the Chinese People’s Liberation Army, Dalian, 116000 People’s Republic of China

**Keywords:** Biomarkers, Diseases

## Abstract

Epidemiological studies have suggested that cold is an important contributor to acute cardiovascular events and mortality. However, little is known about the Diurnal Temperature Range (DTR) impact on mortality of the patients with myocardial infarction. Calcium ions (Ca^2+^) play a vital role in the human body, such as cardiac electrophysiology and contraction. To investigate whether DTR on admission moderates the association between serum calcium and in-hospital mortality in patients with acute myocardial infarction (AMI). This retrospective study enrolled consecutive adult patients with AMI at a single center in China (2003–2012). Patients were divided into four groups (Ca-Q1–4) according to serum calcium concentration quartiles. Multivariate logistic regression modeling was used to assess whether DTR moderated the association between serum calcium and in-hospital mortality. The predictive value of serum calcium was evaluated by receiver operating characteristic (ROC) curve and net reclassification improvement (NRI) analyses. The study included 3780 patients. In-hospital mortality was 4.97% (188/3780). DTR moderated the association between serum calcium and in-hospital mortality (*P-interaction* = 0.020). Patients with low serum calcium in the highest DTR quartile exhibited an increased risk of in-hospital mortality (odds ratio for Ca-Q4 vs. Ca-Q1, 0.03; 95% confidence interval [95% CI], 0.01–0.20). In the highest DTR quartile, adding serum calcium concentration to the risk factor model increased the area under the ROC curve (0.81 vs. 0.76; *P* < 0.001) and increased NRI by 20.2% (95% CI 7.5–32.9; *P* = 0.001). Low serum calcium was an independent risk factor for in-hospital mortality in patients with AMI, and this association was moderated by DTR. Careful attention should be paid to patients with low serum calcium who experience a higher DTR on admission.

## Introduction

Ischemic heart disease (IHD) is a major public health issue and in 2015 was responsible for over 8.9 million deaths globally^1^. The overall prevalence of IHD in the USA was recently reported to be 6.3% and was higher in males (7.4%) than in females(5.3%)^2^. Acute myocardial infarction (AMI) is a major cause of mortality due to IHD^1^.In recent years, research on the relationship between climate change and health has received increasing attention^3,4^.Epidemiological studies have suggested that DTR is a risk factor for acute cardiovascular events and mortality^4–7^. Calcium ions (Ca^2+^) play a vital role in physiological and biochemical processes in the human body. Previous studies have suggested that both low and high levels of serum calcium can increase the incidence of cardiovascular disease and all-cause mortality^8–10^. However, the results are inconsistent between populations with differing characteristics, especially between patients with differing diseases^8,11^.Experimental studies in animals have shown that calcium and calpain metabolism are disturbed at low temperatures and that exposure to cold temperatures results in elevated intracellular calcium concentrations^12^.We hypothesized that the relationship between serum calcium concentration and in-hospital mortality in patients with AMI may be moderated by the admission DTR. Therefore, the objective of this study was to evaluate the potential moderating effect of admission DTR on the association between serum calcium concentration and in-hospital mortality in a 10-year cohort of patients with AMI from north-eastern China.

## Material and methods

### Study design and population

This retrospective study enrolled consecutive patients with AMI who were admitted to the Department of Cardiology, First Affiliated Hospital of Harbin Medical University, Harbin, China between January 2003 and December 2012. Our hospital is the largest hospital in Heilongjiang province and serves patients in the local and surrounding areas. Our hospital was not air-conditioned during the period of the study, and hence the temperature within the hospital may have shown variation with outdoor temperature. The inclusion criteria were: age ≥ 18 years; and a clinical diagnosis of AMI(new or recurrent)was made based on the presence of chest pain lasting > 20 min, characteristic alterations in the electrocardiogram(such as new pathological Q waves or ST-segment and T-wave changes), elevated levels of cardiac enzymes and/or imaging evidence of myocardial necrosis. The exclusion criteria were: congenital heart disease; infectious diseases; pregnancy; cancer; psychiatric disorders; and information required for the analysis (including the results of laboratory investigations) was missing. The Human Ethics Review Board at Harbin Medical University and the Ethics Committee of the First Affiliated Hospital of Harbin Medical University approved this study.

### Data collection and outcome measures

A team of trained researchers, including cardiologists, postgraduates and statistical analysts, reviewed the eligibility of the study participants and retrospectively collected clinical data from the medical records, including patient admission time, demographics, medical histories, diagnosis-related information and results of laboratory investigations. Where possible, missing information was obtained by face-to-face interviews with the patients or their family members. Hypertension was defined as a systolic blood pressure ≥ 140 mm Hg and/or a diastolic blood pressure ≥ 90 mm Hg or use of antihypertensive medications. Current smoking was defined as smoking at least one cigarette per day during the previous year^19^. Previous stroke and diabetes mellitus were identified from each patient’s clinical history and confirmed by consultation with an experienced neurologist or endocrinologist. Venous blood samples from each patient were obtained within the first 24 h of admission to the coronary care unit. The laboratory investigations, including measurement of serum calcium level, were carried out at our hospital’s clinical laboratories. Plasma ion concentrations were measured using an ion-specific electrode (Auto Analyzer; Hitachi Inc., Tokyo, Japan).The levels of plasma total cholesterol, total triglycerides, high-density lipoprotein cholesterol (HDL-C), low-density lipoprotein cholesterol (LDL-C), fasting glucose, serum creatinine, uric acid and blood urea nitrogen (BUN)were analyzed using an automatic biochemical analyzer (Beckman Coulter, Brea, CA, USA).Daily meteorological data, such as the minimum, maximum and mean temperature, for the period January 2003 to December 2012 were obtained from the China Meteorological Data Sharing System. For each patient, the DTR on the day of admission was obtained by subtracting the minimum temperature on that day from the maximum temperature. The main outcome measure of our study was all-cause in-hospital mortality.

### Statistical analysis

The study participants were divided into 4 groups according to the quartiles of admission serum calcium concentration:Ca-Q1 (< 2.19 mmol/L), Ca-Q2(2.19–2.26 mmol/L), Ca-Q3 (2.27–2.33 mmol/L)and Ca-Q4 (> 2.33 mmol/L).Data distribution was examined using the Kolmogorov–Smirnov test. Normally distributed continuous variables are presented as the mean (standard deviation, SD) and were compared between groups using one-way analysis of variance (ANOVA)and the Bonferroni post-hoc test. Non-normally distributed continuous variables are presented as median (interquartile range, IQR)and were compared between groups using the Kruskal–Wallis test. Categorical variables are presented as proportions (%) and were analyzed using the chi-squared test. Logistic regression was used to assess the independent relationship between serum calcium level and in-hospital mortality of patients with AMI, with adjustments for other potential confounders. First, univariate analysis was performed to identify factors associated with in-hospital mortality. Then, to confirm an independent association between serum calcium concentration and in-hospital mortality, we established three multivariate logistic regression models: model 1 (unadjusted), model 2 (adjusted for age and gender) and model 3 (adjusted for all factors with *P* < 0.05 in the univariate analysis). Serum calcium concentration was included in each of the three regression models as quartile categories and as a continuous variable (in separate analyses). Next, the association between DTR and in-hospital mortality was investigated using the three multivariate logistic regression models: model 1 (unadjusted), model 2 (adjusted for age and gender) and model 3 (adjusted for all factors with *P* < 0.05 in the univariate analysis). DTR was included in each of the three regression models as quartile categories (DTR-Q1: < 7.7 °C; DTR-Q2: 7.7–9.9 °C; DTR-Q3: 10.0–12.6 °C; and DTR-Q4: > 12.6 °C) and as a continuous variable (in separate analyses).To establish whether DTR might moderate the effects of other factors on in-hospital mortality, subgroup analyses were performed stratified by age, gender, current smoking status, current alcohol use, history of hypertension, history of diabetes mellitus, angiotensin converting enzyme inhibitor (ACEI)/angiotensin receptor blocker (ARB) use, beta-blocker usetype of AMI and DTR. Smooth curve fitting was used to assess the relationships between serum calcium concentration and in-hospital mortality stratified by the quartiles of DTR. The independent predictive value of serum calcium concentration for different quartiles of DTR was examined by comparing the area under the receiver operator characteristic curve (ROC_AUC_).The multivariate combined predictive value of serum calcium concentration was estimated using a regression model that included the factors associated with in-hospital mortality in our study with or without serum calcium. Net reclassification improvement (NRI) was determined to calculate the percentage of individuals with or without in-hospital death that would be correctly reclassified when the existing model was updated by the inclusion of serum calcium concentration. R software (version 4.05) was used for data management, generating plots and statistical analyses. The NRI was analyzed using the R package, “PredictABEL”^13^. Two-sided *P* < 0.05 was considered statistically significant for all analyses.

## Results

### Baseline demographic and clinical characteristics of the study participants stratified by serum calcium concentration on admission

A total of 3780 patients with AMI met the criteria for inclusion in our study. Among these 3780 patients, 188 (4.97%) died during hospitalization. The mean age of all our study patients was 58.9 years (SD, 10.6 years), and 2798 patients (74.0%) were male. A total of 1608 patients (42.5%) had hypertension, 759 (20.1%) had a history of diabetes mellitus, and 311 (8.2%) had a history of stroke. Most patients had STEMI (2614/3780, 69.2%), and more than half were current smokers (2030/3780, 53.7%).

Serum calcium concentration (measured in each patient at the time of hospital admission) approximated to a normal distribution (Supplemental Fig. S1) with a mean level of 2.26 mmol/L (SD, 0.15 mmol/L) and a median level of 2.26 mmol/L (IQR, 2.19–2.33 mmol/L). The distribution of DTR exhibited slight positive skewness (Supplemental Figure S2) with a mean temperature difference of 10.26 °C (SD, 3.56 °C) and a median temperature difference of 9.90 °C (IQR, 7.69–12.60 °C).

Based on the admission serum calcium concentrations, the patients were stratified into quartiles: Ca-Q1 (serum calcium concentration < 2.19 mmol/L), *n* = 892; Ca-Q2 (serum calcium concentration 2.19–2.26 mmol/L), *n* = 997; Ca-Q3 (serum calcium concentration 2.27–2.33 mmol/L), *n* = 883; and Ca-Q4 (serum calcium concentration > 2.33 mmol/L), *n* = 1008. The clinical characteristics of the patients in each of the serum calcium quartiles are shown in Table 1. Age, BUN, HDL-C, serum creatinine, uric acid, serum phosphate, serum magnesium, serum chloride and left atrial diameter (LAD) were significantly higher in patients with low serum calcium levels than in patients with elevated levels of serum calcium (all *P* < 0.05; Table 1).

**Table 1:** Clinical characteristics of the 3780 patients with acute myocardial infarction stratified according to serum calcium concentration quartiles.

Characteristic	Serum calcium concentration quartile				
	Ca-Q1 (*n* = 892)	Ca-Q2 (*n* = 997)	Ca-Q3 (*n* = 883)	Ca-Q4 (*n* = 1008)	*P*
**Demographics**					
Age, years	63.0 (55.0–70.0)	61.0 (53.0–69.0)	58.0 (50.0–67.0)	57.0 (48.0–66.0)	< 0.001
Men (*n,* %)	650 (72.9%)	738 (74.0%)	651 (73.7%)	759 (75.3%)	0.680
Current smoking (*n,* %)	473 (53.0%)	546 (54.8%)	448 (50.7%)	563 (55.9%)	0.132
Current alcohol use (*n*, %)	90 (10.1%)	105 (10.5%)	98 (11.1%)	132 (13.1%)	0.159
**Medical history**					
Hypertension (*n*, %)	268 (30.0%)	416 (41.7%)	418 (47.3%)	506 (50.2%)	< 0.001
Diabetes mellitus (*n*, %)	156 (17.5%)	208 (20.9%)	184 (20.8%)	211 (20.9%)	0.180
Stroke (*n*, %)	84 (9.4%)	77 (7.7%)	71 (8.0%)	79 (7.8%)	0.522
Hyperlipidemia (*n*, %)	68 (7.6%)	141 (14.1%)	174 (19.7%)	206 (20.4%)	< 0.001
**In-hospital complications**					
Acute heart failure (*n*, %)	97 (10.9%)	134 (13.4%)	117 (13.3%)	133 (13.2%)	0.304
Acute arrhythmia (*n*, %)	30 (3.4%)	44 (4.4%)	38 (4.3%)	47 (4.7%)	0.528
**Medication on admission**					
ACEI/ARB (*n*, %)	445 (49.9%)	497 (49.8%)	462 (52.3%)	500 (49.6%)	0.621
Beta-blocker (*n*, %)	511 (57.3%)	569 (57.1%)	521 (59.0%)	560 (55.6%)	0.514
Aspirin (*n*, %)	621 (69.6%)	726 (72.8%)	621 (70.3%)	703 (69.7%)	0.367
Statin (*n*, %)	713 (79.9%)	768 (77.0%)	708 (80.2%)	780 (77.4%)	0.205
**Main diagnosis**					
NSTEMI (*n*, %)	280 (31.4%)	308 (30.9%)	266 (30.1%)	312 (31.0%)	0.168
STEMI (*n*, %)	612 (68.6%)	689 (69.1%)	617 (69.9%)	696 (69.0%)	0.025
**Reperfusion strategy**					
PCI (*n*, %)	345 (38.7%)	431 (43.2%)	358 (40.5%)	404 (40.1%)	0.228
Thrombolysis (*n*, %)	191 (21.4%)	215 (21.6%)	203 (23.0%)	202 (20.0%)	0.486
**Laboratory results**					
Albumin (g/L)	37.4 (34.9–39.8)	38.5 (36.5–40.9)	39.3 (37.0–41.6)	40.1 (37.7–42.6)	0.651
BUN (mmol/L)	6.0 (4.8–7.6)	5.8 (4.8–7.0)	5.7 (4.6–6.8)	5.5 (4.6–6.9)	0.017
Total cholesterol (mmol/L)	4.4 (3.6–5.2)	4.5 (3.7–5.2)	4.7 (3.7–5.7)	4.8 (3.9–5.8)	0.517
Total triglycerides (mmol/L)	1.6 (1.0–2.4)	1.8 (1.2–3.2)	2.0 (1.3–3.7)	2.0 (1.3–3.3)	0.674
HDL-C (mmol/L)	1.2 (1.0–1.4)	1.2 (1.1–1.4)	1.3 (1.1–1.5)	1.2 (1.0–1.4)	0.032
LDL-C (mmol/L)	3.0 (2.4–3.7)	3.2 (2.6–3.8)	3.3 (2.8–3.9)	3.4 (2.7–4.1)	0.766
Fasting glucose (mmol/L)	8.0 (6.3–11.0)	7.9 (6.3–10.5)	7.9 (6.5–10.2)	7.7 (6.4–10.5)	0.408
Serum creatinine (µmol/L)	81.0 (68.4–97.9)	81.1 (69.2–94.5)	79.8 (70.1–92.3)	78.0 (68.7–91.0)	< 0.001
Uric acid (µmol/L)	305.5 (256.5–367.4)	314.4 (272.4–366.4)	317.4 (276.7–363.5)	323.7 (276.0–382.5)	< 0.001
Serum phosphate (mmol/L)	1.1 (0.9–1.4)	1.1 (1.0–1.2)	1.1 (1.0–1.2)	1.1 (1.0–1.2)	0.028
Serum magnesium (mmol/L)	0.9 (0.8–1.0)	0.9 (0.8–0.9)	0.9 (0.8–0.9)	0.9 (0.8–0.9)	0.028
Serum potassium (mmol/L)	3.9 (3.6–4.3)	4.0 (3.7–4.3)	4.0 (3.7–4.2)	4.0 (3.8–4.3)	0.055
Serum sodium (mmol/L)	137.6 (134.7–140.1)	138.3 (135.7–141.0)	138.8 (136.0–141.4)	139.2 (136.3–141.7)	0.463
Serum chloride (mmol/L)	101.8 (99.0–104.2)	101.7 (99.3–104.2)	101.6 (99.4–103.8)	101.5 (98.9–104.0)	0.018
**Echocardiography results**					
LVEF (%)	49.0 (41.0–56.0)	50.0 (43.0–56.0)	50.0 (43.6–56.0)	51.0 (44.2–56.9)	0.010
LAD (mm)	37.0 (35.0–40.0)	37.0 (34.8–39.0)	36.7 (34.7–39.0)	36.4 (34.0–39.0)	0.047
LVEDD (mm)	51.0 (47.6–54.0)	50.8 (47.0–53.9)	50.0 (47.0–53.5)	50.0 (47.0–53.0)	0.248
**Temperature information**					
Daily maximum temperature	11.42 (14.91)	11.32 (14.61)	9.61 (14.85)	9.19 (15.21)	< 0.001
Daily minimum temperature	1.05 (14.30)	1.05 (14.05)	−0.49 (14.19)	−1.12 (14.70)	< 0.001
Daily mean temperature	6.09 (14.57)	6.08 (14.26)	4.44 (14.47)	3.92 (14.89)	< 0.001
**Main index and outcome measure**					
DTR ( °C)	10.0 (7.6–12.8)	9.9 (7.7–12.5)	9.8 (7.5–12.4)	10.0 (7.8–12.6)	0.426
In-hospital mortality (*n*, %)	84 (9.4%)	46 (4.6%)	43 (4.9%)	15 (1.5%)	< 0.001

Data are expressed as median (interquartile range) or *n* (%). Ca-Q1: serum calcium concentration < 2.19 mmol/L; Ca-Q2: serum calcium concentration 2.19–2.26 mmol/L; Ca-Q3: serum calcium concentration 2.27–2.33 mmol/L; Ca-Q4: serum calcium concentration > 2.33 mmol/L. Abbreviations: ACEI, angiotensin converting enzyme inhibitor; ARB, angiotensin receptor blocker; BUN, blood urea nitrogen; DTR, diurnal temperature range; HDL-C, high-density lipoprotein cholesterol; LAD, left atrial diameter; LDL-C, low-density lipoprotein cholesterol; LVEDD, left ventricular end-diastolic diameter; LVEF, left ventricular ejection fraction; NSTEMI, non-ST-segment elevation myocardial infarction; PCI, percutaneous coronary intervention; STEMI, ST-segment elevation myocardial infarction.

### Association between serum calcium concentration and in-hospital mortality

**Figure 1:**
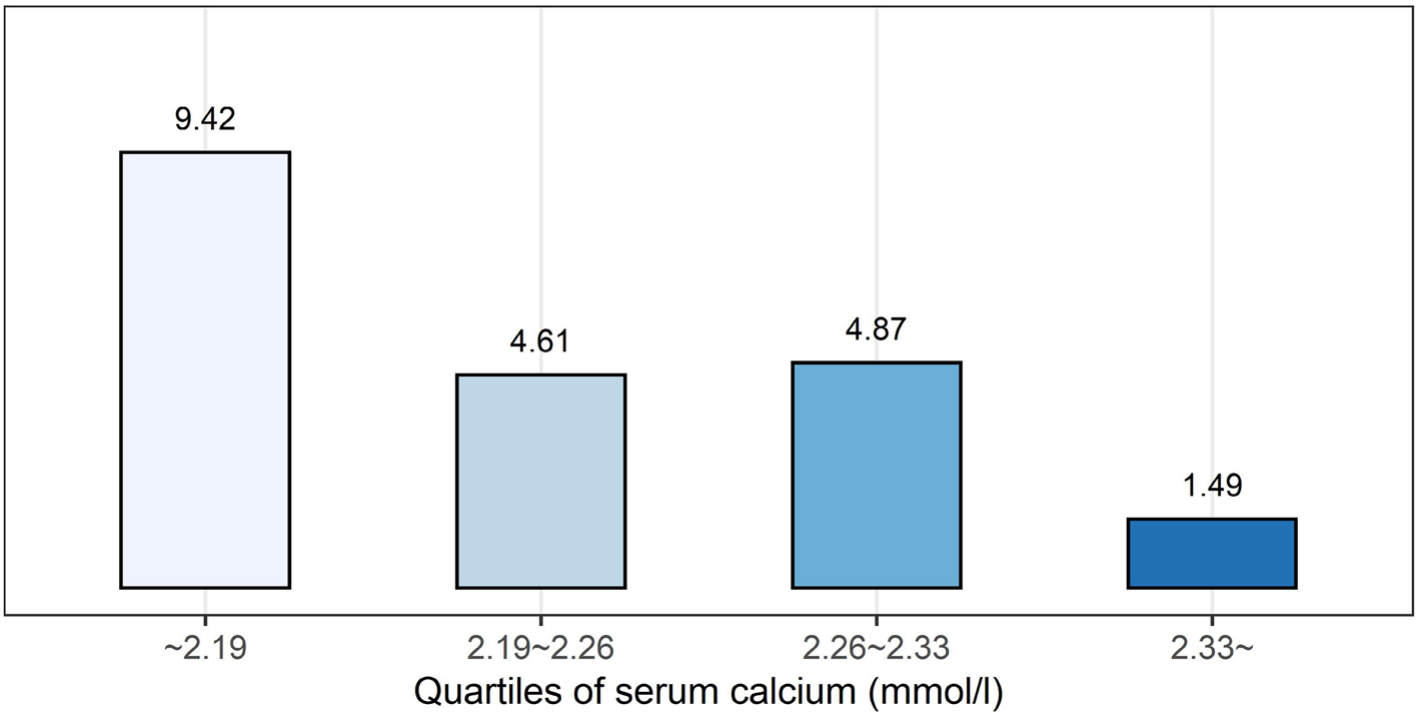
In-hospital mortality of patients with AMI according to the quartile of the distribution of serum calcium concentration on admission. In-hospital mortality increased with a decrease in the quartile of serum calcium concentration.

Patients in the lowest serum calcium quartile exhibited the highest incidence of in-hospital mortality (Table 1). As shown in Fig. 1, in-hospital mortality progressively increased with a decrease in the quartile of serum calcium concentration.

Univariate logistic regression analysis found that age, gender, current smoking, current alcohol use, diabetes mellitus, HDL-C, uric acid, serum phosphate, serum potassium, left ventricular ejection fraction (LVEF), LAD and serum calcium concentration were factors significantly associated with in-hospital mortality (Supplemental Table S1). However, multivariate logistic regression analysis revealed that only age, gender, diabetes mellitus, HDL-C, uric acid, serum phosphate, LVEF and serum calcium concentration were independently associated with in-hospital mortality (Supplemental Table S2).

As shown in Table 2, the odds of in-hospital mortality were significantly lower among study patients with serum calcium concentration in the highest quartile (> 2.33 mmol/L) than among those with serum calcium concentration in the lowest quartile (< 2.19 mmol/L) in model 1 (unadjusted; OR, 0.53; 95% CI, 0.45–0.63; *P* for trend < 0.001), model 2 (adjusted for age and gender; OR, 0.57; 95% CI, 0.48–0.68; *P* for trend < 0.001) and model 3 (adjusted for all significant factors in the univariate analysis; OR, 0.56; 95% CI, 0.47–0.67; *P* for trend < 0.001).

**Table 2:** The association between serum calcium concentration and in-hospital mortality of patients with acute myocardial infarction analyzed using three different logistic regression models.

Model	Serum calcium concentration			
	Ca-Q1	Ca-Q2	Ca-Q3	Ca-Q4
Deaths/*N*	84/892	46/951	43/840	15/993
Model 1	Ref	0.47 (0.32–0.68) *P* < 0.001	0.49 (0.34–0.72) *P* < 0.001	0.15 (0.08–0.25) *P* < 0.001
Model 2	Ref	0.49 (0.34–0.71) *P* < 0.001	0.56 (0.38–0.82) *P* = 0.003	0.17 (0.10–0.31) *P* < 0.001
Model 3	Ref	0.47 (0.32–0.69) *P* < 0.001	0.55 (0.37–0.82) *P* = 0.003	0.14 (0.07–0.25) *P* < 0.001

Data are presented as odds ratio (95% confidence interval), *P* value. Ca-Q1: serum calcium concentration < 2.19 mmol/L; Ca-Q2: serum calcium concentration 2.19–2.26 mmol/L; Ca-Q3: serum calcium concentration 2.27–2.33 mmol/L; Ca-Q4: serum calcium concentration > 2.33 mmol/L. Model 1: unadjusted. Model 2: adjusted for age and gender. Model 3: adjusted for age, gender, current smoking, current alcohol use, diabetes mellitus, high-density lipoprotein cholesterol, uric acid, serum phosphate, serum potassium, left ventricular ejection fraction and left atrial diameter.

### Moderating effect of DTR on the association between serum calcium concentration and in-hospital mortality

The patients were stratified into quartiles based on admission DTR: DTR-Q1 (DTR < 7.7 °C), *n* = 945; DTR-Q2 (DTR 7.7–9.9 °C), *n* = 910; DTR-Q3 (DTR 10.0–12.6 °C), *n* = 972; and DTR-Q4 (DTR > 12.6 °C), *n* = 953. Logistic regression analysis showed that DTR was not independently associated with in-hospital mortality (Supplemental Table S3). However, subgroup analyses revealed that the association between serum calcium concentration and in-hospital mortality was moderated by different quartiles of admission DTR (Table 3). Although the association between serum calcium concentration and in-hospital mortality was significant in most of the subgroups analyzed (Table 3), only DTR was observed to interact significantly with serum calcium concentration (*P-interaction* = 0.020).

**Table 3:** The association between serum calcium concentration and in-hospital mortality of patients with acute myocardial infarction: subgroup analyses.

Model	Serum calcium concentration				
	Ca-Q1	Ca-Q2	Ca-Q3	Ca-Q4	*P-interaction*
**Age**					0.519
< 60 years	Ref	0.24 (0.09–0.66)	0.60 (0.30–1.23)	0.17 (0.06–0.46)	
≥ 60 years	Ref	0.53 (0.34–0.82)	0.48 (0.29–0.79)	0.11 (0.05–0.24)	
**Gender**					0.289
Male	Ref	0.86 (0.46–1.61)	0.90 (0.47–1.70)	0.12 (0.04–0.36)	
Female	Ref	0.33 (0.19–0.56)	0.41 (0.24–0.70)	0.15 (0.07–0.31)	
**Current smoker**					0.582
No	Ref	0.71 (0.43–1.18)	0.71 (0.42–1.19)	0.08 (0.03–0.20)	
Yes	Ref	0.24 (0.12–0.48)	0.39 (0.20–0.76)	0.19 (0.08–0.44)	
**Current alcohol use**					0.858
No	Ref	0.46 (0.31–0.69)	0.54 (0.36–0.81)	0.15 (0.08–0.27)	
Yes	Ref	0.68 (0.10–4.57)	0.88 (0.14–5.58)	NA	
**History of hypertension**					0.963
No	Ref	0.38 (0.22–0.67)	0.48 (0.28–0.83)	0.16 (0.08–0.36)	
Yes	Ref	0.62 (0.35–1.10)	0.36 (0.36–1.20)	0.08 (0.03–0.24)	
**History of diabetes**					0.190
No	Ref	0.39 (0.24–0.64)	0.52 (0.32–0.84)	0.09 (0.04–0.23)	
Yes	Ref	0.71 (0.35–1.44)	0.66 (0.31–1.41)	0.24 (0.10–0.58)	
**ACEI/ARB**					0.263
No	Ref	0.56 (0.32–0.99)	0.75 (0.43–1.32)	0.12 (0.05–0.31)	
Yes	Ref	0.41 (0.23–0.71)	0.42 (0.24–0.76)	0.15 (0.07–0.34)	
**Beta-blocker**					0.542
No	Ref	0.62 (0.35–1.08)	0.55 (0.29–1.02)	0.20 (0.09–0.46)	
Yes	Ref	0.38 (0.21–0.66)	0.59 (0.35–0.99)	0.08 (0.03–0.21)	
**Type of AMI**					0.263
NSTEMI	Ref	0.28 (0.13–0.59)	0.58 (0.30–1.13)	0.06 (0.02–0.23)	
STEMI	Ref	0.61 (0.39–0.98)	0.59 (0.36–0.97)	0.19 (0.10–0.38)	
**DTR**					0.020
DTR-Q1	Ref	0.55 (0.22–1.36)	0.57 (0.23–1.45)	0.44 (0.16–1.24)	
DTR-Q2	Ref	0.65 (0.28–1.48)	0.50 (0.21–1.23)	0.16 (0.05–0.55)	
DTR-Q3	Ref	0.63 (0.30–1.30)	0.86 (0.39–1.86)	0.14 (0.04–0.50)	
DTR-Q4	Ref	0.23 (0.10–0.53)	0.44 (0.21–0.92)	0.03 (0.01–0.20)	

All analyses were adjusted for the same variables as model 3 in Table 2, except for the stratification variable. Ca-Q1: serum calcium concentration < 2.19 mmol/L; Ca-Q2: serum calcium concentration 2.19–2.26 mmol/L; Ca-Q3: serum calcium concentration 2.27–2.33 mmol/L; Ca-Q4: serum calcium concentration > 2.33 mmol/L; DTR-Q1: diurnal temperature range < 7.7 °C; DTR-Q2: diurnal temperature range 7.7–9.9 °C; DTR-Q3: diurnal temperature range 10.0–12.6 °C; DTR-Q4: diurnal temperature range > 12.6 °C. NA: not available due to limited sample size. Abbreviations: ACEI, angiotensin converting enzyme inhibitor; AMI, acute myocardial infarction; ARB, angiotensin receptor blocker; DTR, diurnal temperature range; CI, confidence interval; NSTEMI, non-ST-segment elevation myocardial infarction; OR, odds ratio; STEMI, ST-segment elevation myocardial infarction.

**Figure 2:**
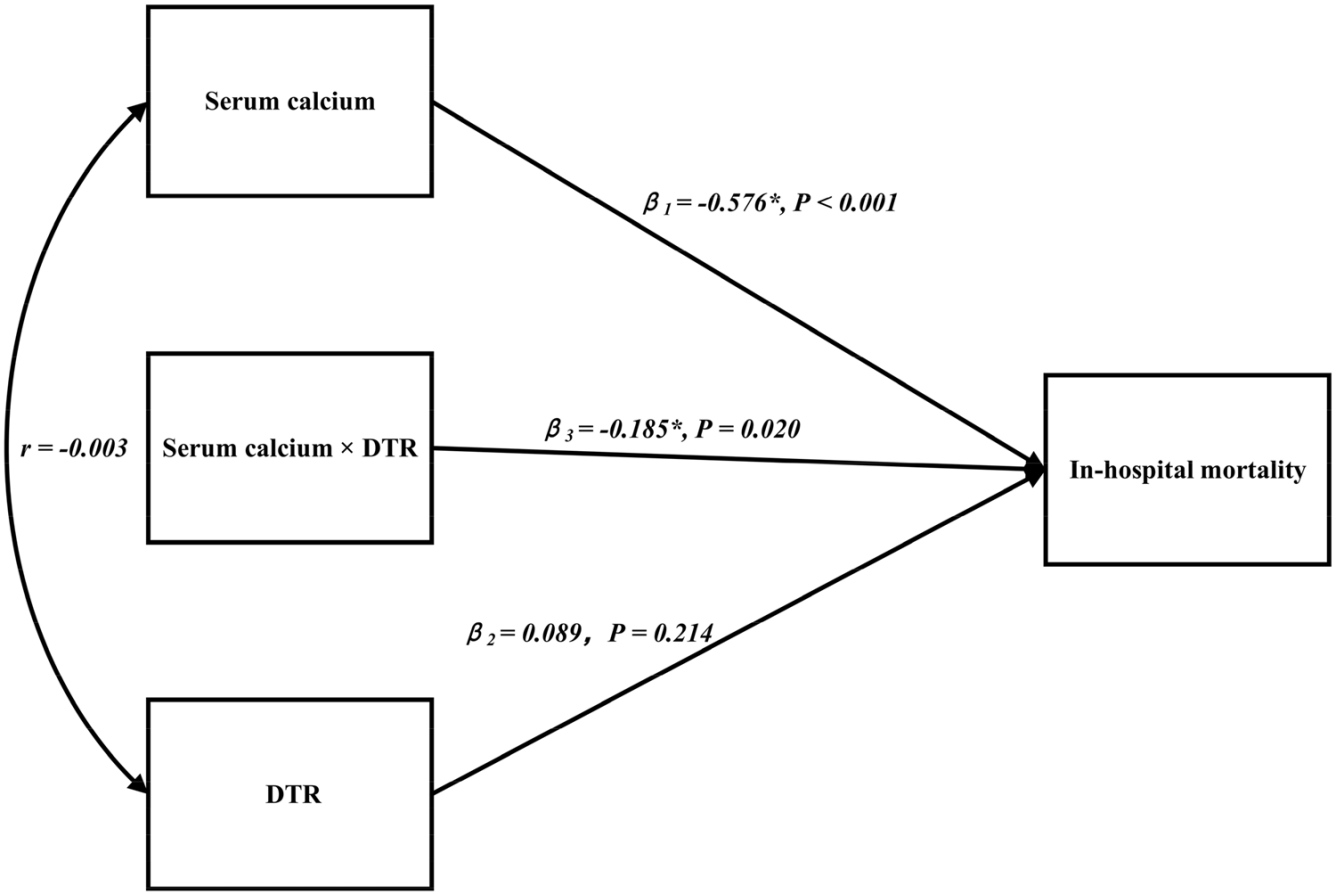
Model to show how different quartiles of admission diurnal temperature range (DTR) moderated the association between serum calcium concentration and in-hospital mortality of patients with acute myocardial infarction. *β*_*1*_ = age, gender, current smoking, current alcohol use, diabetes mellitus, high-density lipoprotein cholesterol, uric acid, serum phosphate, serum potassium, left ventricular ejection fraction and left atrial diameter. *β*_*2*_ = the independent effect of diurnal temperature range (DTR) on in-hospital mortality after adjusting for the above covariates. *β*_*3*_ = the interaction effect of serum calcium concentration and DTR on in-hospital mortality after adjusting for the above covariates. *r* = correlation between serum calcium concentration and DTR. *P < 0.05.

**Figure 3:**
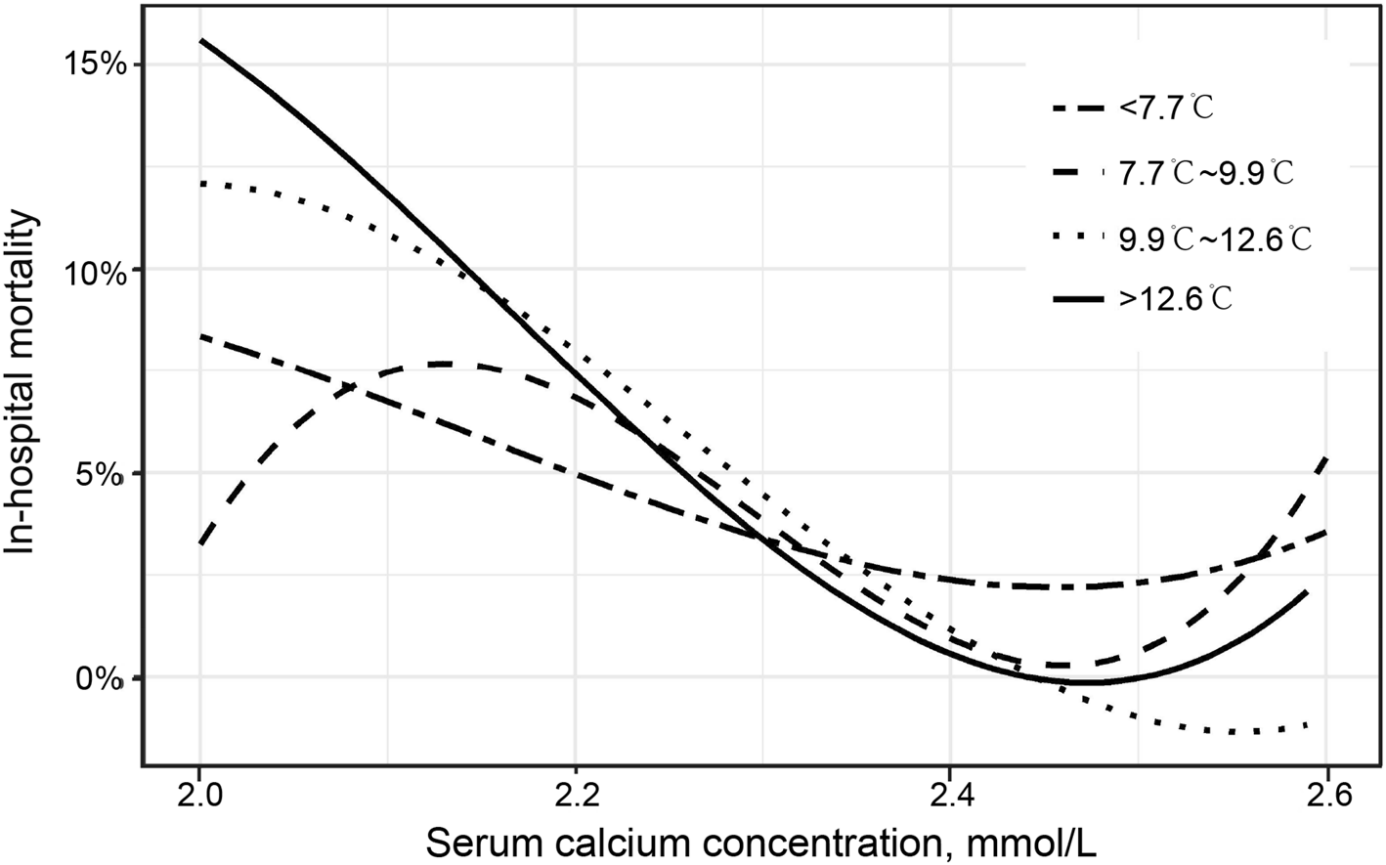
Spline plots displaying the risk of in-hospital mortality over a range of serum calcium concentrations stratified by quartiles of diurnal temperature range (DTR). Smooth curve fitting revealed an L-shaped relationship of in-hospital mortality with serum calcium concentration for the third and fourth quartiles of DTR.

Supplemental Table 4 showed that patients with low serum calcium levels in the highest quartile of admission DTR (> 12.6 °C) had a notably increased risk of in-hospital mortality compared with patients in the lowest quartile of DTR (< 7.7 °C) after adjustment for potential confounders (Q4:Q1 OR, 0.04; 95% CI, 0.01–0.23; *P* < 0.001). This strong negative association of DTR with in-hospital mortality was consistent between different regression models and the various quartiles of serum calcium concentration. This model of the moderating effect of DTR, built based on a multivariate logistic regression model, is shown in Fig. 2. The Odds ratios and 95% CIs of in-hospital mortality of serum calcium concentration (Q4 vs. Q1) for patients in the lowest, second and third quartiles of DTR were 0.29 (95% CI, 0.10–0.86; *P* = 0.025), 0.20(95% CI, 0.06–0.69; *P* = 0.011), and 0.15 (95% CI, 0.04–0.55; *P* = 0.004) respectively. Smooth curve fitting showed that for the third and fourth quartiles of DTR, the relationship between serum calcium concentration and in-hospital mortality was L-shaped, and the in-hospital mortality progressively decreased with increasing serum calcium concentration up to ~ 2.45 mmol/L (Fig. 3).

**Table 4:** Net reclassification improvement among patients in the highest quartile of diurnal temperature range (> 12.6 °C) after adjustment for serum calcium concentration.

Predicted risk (without serum calcium)	Reclassified predicted risk (with serum calcium)			Reclassified (%)		
	< 15%	15–30%	> 30%	Increased risk	Decreased risk	Net correctly reclassified (%)
**Incidence of in-hospital death**						
< 15%	36	10	0			
15–30%	0	2	3	30.9%	2.4%	28.6%
> 30%	0	1	0	13	1	
**Incidence of survival**						
< 15%	835	31	4			
15–30%	13	9	5	4.4%	1.6%	2.9%
> 30%	1	0	3	40	14	

Net reclassification improvement (categorical) = 20.2% (95% confidence interval, 7.5–32.9; *P* = 0.001). Net reclassification improvement (continuous) = 57.2% (95% confidence interval, 30.0–84.3; *P* < 0.001).

### The value of serum calcium concentration for the prediction of in-hospital mortality

**Figure 4:**
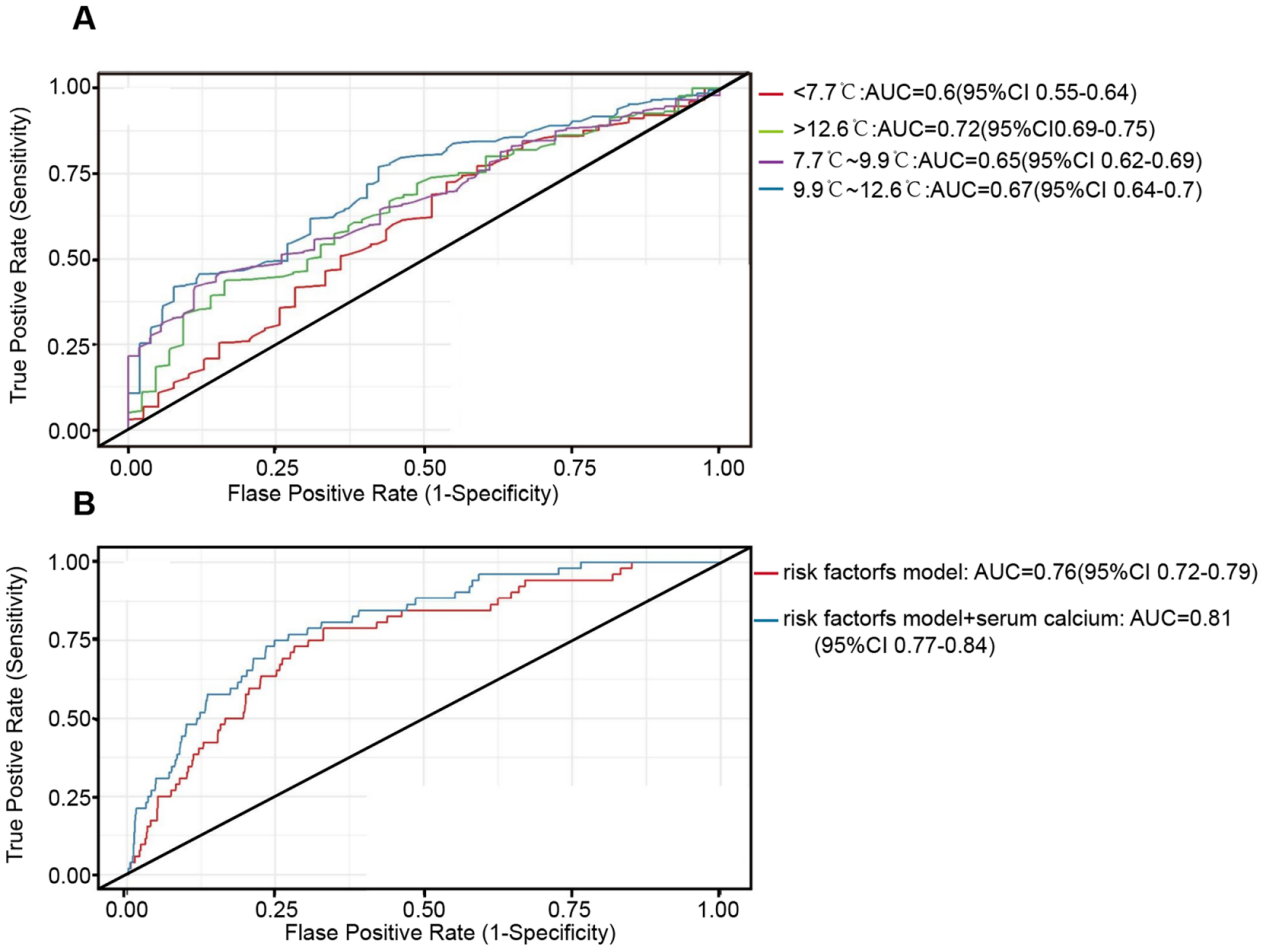
The area under the receiver operating characteristic curve (ROC_AUC_) of serum calcium concentration in the prediction of in-hospital mortality among patients with acute myocardial infarction stratified by quartiles of DTR (A). Compared with other quartiles of DTR, the independent ROC_AUC_ for serum calcium concentration in the highest quartile of DTR was 0.72 (95% CI, 0.69–0.75; *P* < 0.001). Multivariate combined ROC_AUC_, which was estimated using a regression model that included age, gender, current smoking, current alcohol use, diabetes mellitus, high-density lipoprotein cholesterol, uric acid, serum phosphate, serum potassium, left ventricular ejection fraction and left atrial diameter, with or without serum calcium among patients in the highest DTR quartile (B). The combined predictive values with and without serum calcium concentration were 0.81 (95% CI, 0.76–0.84; *P* < 0.001) and 0.76 (95% CI, 0.72–0.79; *P* < 0.001), respectively. AUC, area under the curve; CI, confidence interval.

The value of serum calcium concentration for the prediction of in-hospital mortality in different quartiles of DTR is shown in Fig. 4. Compared with other quartiles of DTR, the independent ROC_AUC_ for serum calcium concentration in the highest quartile of DTR was 0.72 (95% CI, 0.69–0.75; *P* < 0.001). Among patients in the highest quartile of DTR, the combined predictive values in the risk factor models with and without serum calcium concentration were 0.81 (95% CI, 0.76–0.84; *P* < 0.001) and 0.76 (95% CI, 0.72–0.79, *P* < 0.001), respectively.

In addition, we also performed NRI analyses to reveal whether the inclusion of serum calcium concentration would improve the reclassification ability of the model. Among patients in the highest quartile of DTR, the results showed that 2.9% of the individuals who survived and 28.6% of those who died in hospital would be correctly reclassified when the clinical model (including the above risk factors) included serum calcium concentration (Table 4). These reclassification rates led to an estimated NRI of 20.2% (95% CI, 7.5–32.9; *P* = 0.001). In addition, statistically significant NRI was not observed among patients in other quartiles of DTR.

## Discussion

The present study of 3780 patients with AMI demonstrated that low serum calcium level is correlated with increased risk of in-hospital mortality and that this association was moderated by DTR on admission. Patients with low serum calcium levels in the highest quartile of DTR on admission exhibited a notable increase in in-hospital mortality risk. Among the higher quartiles of DTR on admission, we observed an L-shaped relationship between serum calcium concentration and in-hospital mortality. The above association remained consistent with differently adjusted models and with quartiles of serum calcium concentration after adjusting for other potential risk factors. In addition, we observed that the inclusion of serum calcium concentration in the risk factor model could improve the prediction of in-hospital mortality, especially for patients in the highest quartile of admission DTR.

### Serum calcium levels and in-hospital mortality of patients with AMI

Several previous population-based studies have suggested that serum calcium concentration is positively associated with the risk of CHD and all-cause mortality^13–16^. A recent systematic review and meta-analysis of 11 longitudinal studies showed that the hazard ratio (HR) of death was 1.13 per SD of serum calcium concentration among participants with a normal level of serum calcium and no renal disease^17^. A Chinese study first reported that low serum calcium concentration was an independent predictor of in-hospital mortality among patients with STEMI^18^. Our study findings are in agreement with those of the above studies, indicating that low serum calcium levels are an independent predictor of in-hospital mortality in Chinese patients with AMI.

### DTR and in-hospital mortality

Inconsistent with previous studies, our study showed that there was no statistically significant association between DTR and in-hospital mortality. One potential explanation for this apparent discrepancy may be that our study population consisted of patients with AMI whereas the previous studies primarily focused on all hospitalized patients or a general population. A second possible reason is that different areas of China have differing climate characteristics. A recent study reported that mortality is more sensitive to the effects of DTR in the central and southern regions of China than in the northern region^19^. Another report also indicated that the effect of heat-related mortality may be more direct than that of cold-related mortality^20^. A third (and perhaps the main) reason is that our research design was different from those of earlier investigations. Most previous studies used an ecological method in which either exposure or outcome was measured at the group (not individual) level, which can easily lead to an ecological fallacy^21^. A notable advantage of the present study is that our model estimated both the independent moderating effects of meteorological data and the effects of individual cardiovascular risk factors on mortality. With this approach, we observed a clear L-shaped relationship between serum calcium concentration and in-hospital mortality in the highest quartile of admission DTR (> 12.6 °C). The mortality rate of this group markedly increased with a decline of serum calcium level within the normal (2.1–2.6 mmol/L) or abnormal range.

### The modulatory role of DTR

The physiological mechanisms underlying the relationship between low serum calcium concentration and mortality have not been elucidated^7^. Based on our results, we hypothesize that temperature change may play a modulatory role in the mechanism underlying low serum calcium-induced hospitalized death. Several pieces of evidence reported previously might explain this hypothesis. One possible explanation is that a temperature change may affect the function of calcium channels, which could disrupt normal calcium ion levels and ultimately lead to clinical adverse events. Earlier animal studies have reported that exposure to low temperatures could elevate intracellular calcium concentration as well as disturb calpain metabolism and actin disassembly. Another experiment suggested that cold-induced elevation of intracellular calcium concentration could inhibit calcium-activated potassium(BKCa)channels and play a role in the development of hypertension^22^. A low serum calcium concentration aggravates the severity of vitamin D deficiency, thereby affecting cellular growth, proliferation and apoptosis as well as immune system functions; in severe cases, this can cause death^9,23–25^. However, although the mechanisms proposed above may explain the moderating effect of temperature change on the association between serum calcium concentration and mortality, the exact mechanisms remain to be elucidated and require further research.

### Strength and limitations

The strength of our study is that it is the first to investigate the moderating effect of a meteorological factor on the risk of in-hospital mortality among a large patient population. Furthermore, our study included adequate information regarding potential risk factors and confounders and underwent rigorous data quality control. Nevertheless, as with other observational studies, there are still certain limitations. First, although our exposure variable (serum calcium concentration) was measured before the patients reached the primary endpoint, the single-center design and lack of follow-up information limit our ability to determine a causal relationship. Second, the study endpoint focused only on in-hospital mortality. Therefore, our results may not explain the associations with cause-specific death, other clinical outcomes and long-term mortality. Third, previous studies have shown that pre-procedural and post-procedural TIMI flows and acute kidney injury are very important parameter that direct effect the endpoint^26–28^. Fourth, although we have collected as many potential covariates associated with the endpoint as possible, some risk factors (e.g. operational situation and other environmental factors) have not been considered. Fifth, some patients died on their way to hospital or at the time of admission, and this missing information may lead to an underestimation of the associations. Sixth, the duration between symptom onset and admission time for some patients exceeded 24 h. Therefore, the admission DTR and other laboratory indexes may not reflect the exact situations at symptom onset for these patients. Seventh, the study data was collected between 2003 and 2012, during which time the quality of care and prognosis in hospitals may have improved, which may have influenced the study results, but unfortunately, we were not able to collect relevant information in this regard. Finally, although DTR can reflect the temperature change to a certain extent, the temperatures measured at the monitoring station may not represent the actual personal exposure to ambient temperature.

## Conclusions

Low serum calcium concentration is an independent risk factor for in-hospital mortality among patients diagnosed with AMI, and this association may be moderated by admission DTR. In the highest DTR quartile, we observed an obvious L-shaped relationship between serum calcium concentration and in-hospital mortality. Moreover, the inclusion of serum calcium level into the risk factor model may improve the prediction of in-hospital mortality. In our study, the risk of in-hospital mortality appears to increase at serum calcium concentrations that are currently considered to be within the normal range. Serum calcium level may be a possible biomarker that is affected by DTR, and preventive or therapeutic intervention should be provided to patients with low serum calcium concentrations who experience a higher DTR on admission.

## Informed consent

Patient identity remained anonymous, and the requirement for informed consent was waived due to the retrospective observational nature of the study.

## Supplementary Information

Supplementary Information.

## Data Availability

The datasets used and/or analyzed during the current study are available from the corresponding author on reasonable request.
