# Supplementary material for: The synergy between diurnal temperature range and calcium concentration help to predict hospital mortality in patients with acute myocardial infarction
Source: Sci Rep. 2022 Sep 15;12:15527. doi: 10.1038/s41598-022-18816-2 (PMC9477801; doi:10.1038/s41598-022-18816-2)
Supplement: Supplementary file 1 — Supplementary Information. [file 41598_2022_18816_MOESM1_ESM.docx]

**Supplemental Figure 1.** Distribution of serum calcium concentration on admission in the 3780 patients with acute myocardial infarction included in the study. The distribution of serum calcium concentration approximated to a normal distribution with a mean level of 2.26 mmol/L (standard deviation,0.15 mmol/L) and a median level of 2.26 mmol/L (interquartile range,2.19–2.33 mmol/L).


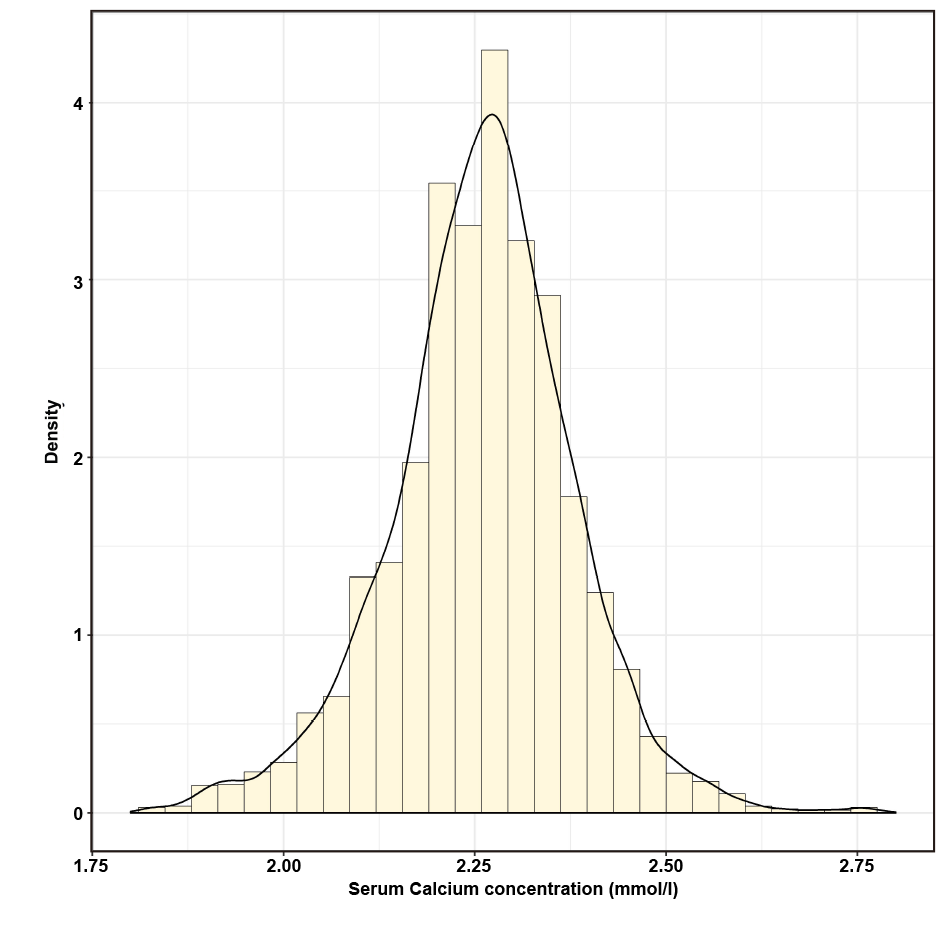


**Supplemental Figure 2.** Distribution of baseline diurnal temperature range on admission in the 3780 patients with acute myocardial infarction included in the study. The distribution of DTR exhibited slight positive skewness with a mean temperature difference of 10.26ºC (standard deviation,3.56ºC) and a median temperature difference of 9.90ºC (interquartile range,7.69–12.60ºC).

**
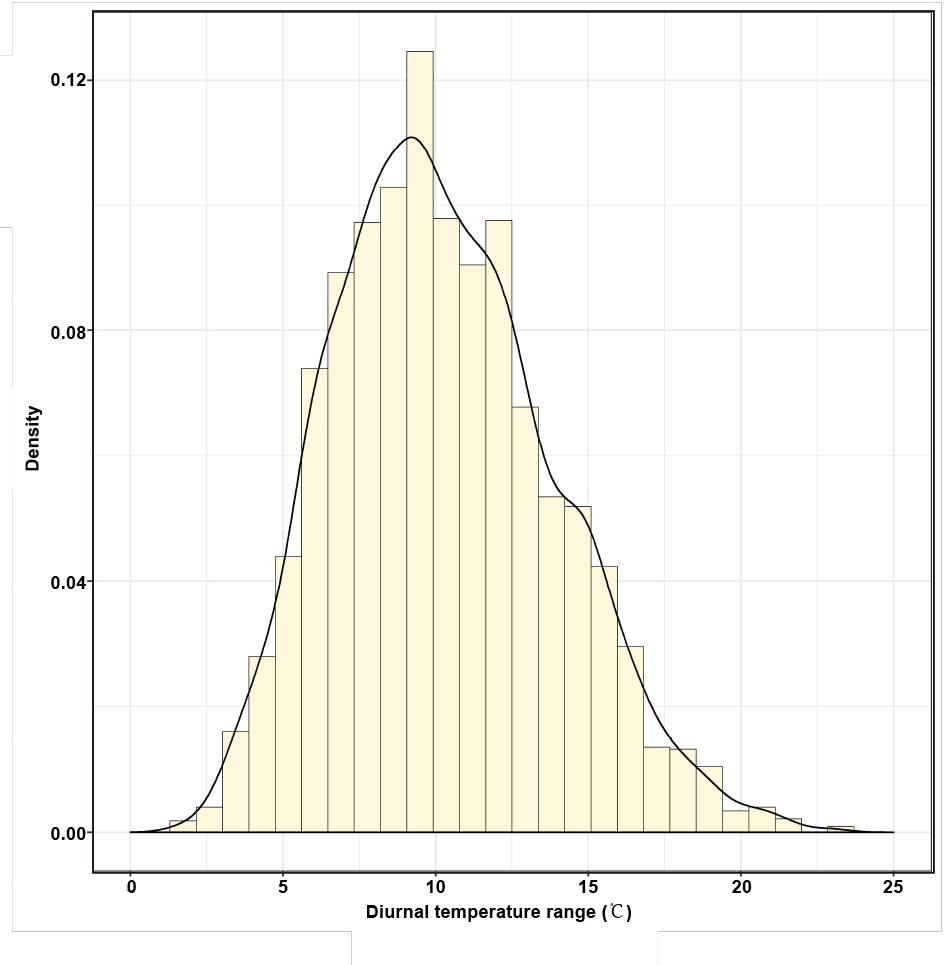
**

**Supplemental Table 1. Univariate analysis of factors associated with in-hospital mortality in patients with acute myocardial infarction.**

| **Factor** | **OR (95%CI)** | ***P*** |
| --- | --- | --- |
| **Demographics** |  |  |
| Age | 1.07 (1.05–1.08) | <0.001 |
| Gender | 0.47 (0.35–0.64) | <0.001 |
| Current smoking | 0.54 (0.40–0.73) | <0.001 |
| Current alcohol use | 0.48 (0.26–0.89) | 0.019 |
| **Medical history** |  |  |
| Hypertension | 0.94 (0.70–1.26) | 0.661 |
| Diabetes mellitus | 2.26 (1.66–3.09) | <0.001 |
| Stroke | 0.89 (0.51–1.56) | 0.690 |
| Hyperlipidemia | 1.30 (0.90–1.89) | 0.168 |
| **In-hospital complications** |  |  |
| Acute heart failure | 1.16 (0.76–1.76) | 0.490 |
| Acute arrhythmia | 1.30 (0.67–2.51) | 0.437 |
| **Medication on admission** |  |  |
| ACEI/ARB | 0.99 (0.73–1.32) | 0.917 |
| Beta-blocker | 0.86 (0.64–1.16) | 0.328 |
| Aspirin | 0.95 (0.69–1.31) | 0.762 |
| Statin | 0.81 (0.58–1.14) | 0.225 |
| **Main diagnosis** |  |  |
| NSTEMI | —— | —— |
| STEMI | 0.86 (0.63–1.17) | 0.331 |
| **Reperfusion strategy** |  |  |
| PCI | 0.86 (0.63–1.16) | 0.323 |
| Thrombolysis | 0.83 (0.57–1.21) | 0.331 |
| **Laboratory results** |  |  |
| Albumin | 1.03 (0.93–1.14) | 0.554 |
| BUN | 1.02 (0.92–1.14) | 0.679 |
| Total cholesterol | 0.91 (0.61–1.36) | 0.651 |
| Total triglycerides | 1.02 (0.92–1.14) | 0.683 |
| HDL-C | 1.08 (1.01–1.17) | 0.036 |
| LDL-C | 1.09 (0.99–1.20) | 0.059 |
| Fasting glucose | 1.07 (1.00–1.15) | 0.051 |
| Serum creatinine | 1.15 (0.99–1.32) | 0.063 |
| Uric acid | 1.47 (1.28–1.69) | <0.001 |
| Serum phosphate | 1.18 (1.10–1.27) | <0.001 |
| Serum magnesium | 1.04 (0.95–1.14) | 0.387 |
| Serum potassium | 1.07 (1.00–1.15) | 0.047 |
| Serum sodium | 1.06 (0.97–1.15) | 0.188 |
| Serum chloride | 0.83 (0.55–1.25) | 0.368 |
| **Echocardiography results** |  |  |
| LVEF | 0.62 (0.53–0.72) | <0.001 |
| LAD | 1.11 (1.03–1.19) | 0.005 |
| LVEDD | 1.01 (0.90–1.14) | 0.823 |
| Temperature information |  |  |
| Daily maximum temperature | 1.00 (0.99, 1.01) | 0.431 |
| Daily minimum temperature | 1.00 (0.99, 1.01) | 0.585 |
| Daily mean temperature | 1.00 (0.99, 1.01) | 0.506 |
| **Main study indexes** |  |  |
| Serum calcium | 0.53 (0.45–0.63) | <0.001 |
| DTR | 1.02 (0.98–1.07) | 0.269 |

Odds ratios were calculated per one standard deviation increment of the continuous variable. Abbreviations: ACEI,angiotensin converting enzyme inhibitor; ARB,angiotensin receptor blocker; BUN,blood urea nitrogen; CI,confidence interval;DTR,diurnal temperature range; HDL-C,high-density lipoprotein cholesterol; LAD,left atrial diameter; LDL-C,low-density lipoprotein cholesterol; LVEDD,left ventricular end diastolic diameter; LVEF,left ventricular ejection fraction; NSTEMI， non-ST-segment elevation myocardial infarction; OR,odds ratio; STEMI,ST-segment elevation myocardial infarction.

**Supplement Table 2. Multivariate analysis of factors independently associated with in-hospital mortality in patients with acute myocardial infarction.**

| **Factor** | ***β*** | ***SE*** | ***z value*** | ***P*** | **OR (95%CI)** |
| --- | --- | --- | --- | --- | --- |
| Age | 0.05 | 0.01 | 5.00 | <0.001 | 1.05 (1.03–1.07) |
| Gender | -0.44 | 0.18 | -2.52 | 0.012 | 0.64 (0.45–0.91) |
| Current smoking | -0.20 | 0.17 | -1.14 | 0.252 | 0.82 (0.58–1.15) |
| Current alcohol use | -0.33 | 0.34 | -0.98 | 0.326 | 0.72 (0.37–1.39) |
| Diabetes mellitus | 0.63 | 0.17 | 3.67 | <0.001 | 1.87 (1.34–2.61) |
| HDL-C | 0.10 | 0.04 | 2.53 | 0.011 | 1.11 (1.02–1.19) |
| Uric acid | 0.45 | 0.08 | 5.59 | <0.001 | 1.57 (1.34–1.85) |
| Serum phosphate | 0.13 | 0.04 | 3.49 | <0.001 | 1.14 (1.06–1.22) |
| Serum potassium | 0.04 | 0.04 | 0.89 | 0.372 | 1.04 (0.95–1.13) |
| LVEF | -0.43 | 0.08 | -5.08 | <0.001 | 0.65 (0.55–0.77) |
| LAD | 0.06 | 0.04 | 1.32 | 0.188 | 1.06 (0.97–1.16) |
| Serum calcium | -0.58 | 0.09 | -6.44 | <0.001 | 0.56 (0.47–0.67) |

Odds ratios were calculated per one standard deviation increment of the continuous variable. Abbreviations: CI,confidence interval; HDL-C, high-density lipoprotein cholesterol; LAD,left atrial diameter; LVEF,left ventricular ejection fraction; OR， odds ratio; SE,standard error.

**Supplemental Table 3. The association between diurnal temperature range and in-hospital mortality of patients with acute myocardial infarction analyzed using three different logistic regression models.**

| Model | Diurnal temperature range | | | |
| --- | --- | --- | --- | --- |
|  | DTR-Q1  (*n* = 945) | DTR-Q2  (*n* = 910) | DTR-Q3  (*n* = 972) | DTR-Q4  (*n* = 953) |
| Model 1 | Ref | 1.15 (0.74–1.80)  *P* = 0.531 | 1.37 (0.90–2.08)  *P* = 0.147 | 1.34 (0.88–2.05)  *P* = 0.177 |
| Model 2 | Ref | 1.14 (0.73–1.78)  *P* = 0.576 | 1.32 (0.86–2.03)  *P* = 0.190 | 1.28 (0.83–1.96)  *P* = 0.265 |
| Model 3 | Ref | 1.16 (0.72–1.85)  *P* = 0.539 | 1.32 (0.84–2.06)  *P* = 0.226 | 1.30 (0.82–2.04)  *P* = 0.256 |

Data are presented as odds ratio (95% confidence interval), *P* value. DTR-Q1: diurnal temperature range <7.7ºC; DTR-Q2: diurnal temperature range 7.7–9.9ºC; DTR-Q3: diurnal temperature range 10.0–12.6ºC; DTR-Q4: diurnal temperature range>12.6ºC. Model 1: unadjusted. Model 2: adjusted for age and gender. Model 3: adjusted for age, gender, current smoking, current alcohol use, diabetes mellitus, high-density lipoprotein cholesterol, uric acid, serum phosphate, serum potassium, left ventricular ejection fraction, left atrial diameter and serum calcium. When serum calcium concentration was analyzed as a continuous variable, the odds ratio was calculated per one standard deviation increment of serum calcium level.

**Supplemental Table 4.**Odds ratios and 95% CIs of in-hospital mortality according to quartiles of serum calcium concentration stratified by quartiles of DTR among patients with acute myocardial infarction.

| Serum calcium | Diurnal temperature range (℃) | | | |
| --- | --- | --- | --- | --- |
|  | Q1: <7.7℃ | Q2: 7.7℃~9.9℃ | Q3: 9.9℃~12.6℃ | Q4: >12.6℃ |
|  | OR (95% CI) *P* | OR (95% CI) *P* | OR (95% CI) *P* | OR (95% CI) *P* |
| Model 1 Q1 | Ref | Ref | Ref | Ref |
| Q2 | 0.49 (0.21, 1.14) 0.097 | 0.60 (0.28, 1.30) 0.196 | 0.65 (0.33, 1.27) 0.205 | 0.23 (0.10, 0.52) <0.001 |
| Q3 | 0.45 (0.19, 1.07) 0.072 | 0.58 (0.26, 1.30) 0.184 | 0.56 (0.27, 1.15) 0.115 | 0.43 (0.21, 0.86) 0.017 |
| Q4 | 0.34 (0.13, 0.89) 0.027 | 0.19 (0.06, 0.57) 0.003 | 0.11 (0.03, 0.36) <0.001 | 0.05 (0.01, 0.23) <0.001 |
| Model 2 Q1 | Ref | Ref | Ref | Ref |
| Q2 | 0.55 (0.23, 1.29) 0.168 | 0.60 (0.26, 1.35) 0.215 | 0.72 (0.36, 1.44) 0.352 | 0.21 (0.09, 0.48) <0.001 |
| Q3 | 0.50 (0.21, 1.22) 0.128 | 0.53 (0.22, 1.25) 0.146 | 0.70 (0.33, 1.49) 0.351 | 0.46 (0.22, 0.93) 0.031 |
| Q4 | 0.41 (0.15, 1.09) 0.073 | 0.21 (0.07, 0.65) 0.007 | 0.13 (0.04, 0.46) 0.002 | 0.06 (0.01, 0.26) <0.001 |
| Model 3 Q1 | Ref | Ref | Ref | Ref |
| Q2 | 0.45 (0.18, 1.17) 0.101 | 0.53 (0.21, 1.35) 0.184 | 0.70 (0.33, 1.51) 0.366 | 0.20 (0.09, 0.46) <0.001 |
| Q3 | 0.50 (0.20, 1.29) 0.152 | 0.57 (0.22, 1.46) 0.240 | 0.86 (0.38, 1.95) 0.726 | 0.45 (0.22, 0.94) 0.033 |
| Q4 | 0.29 (0.10, 0.86) 0.025 | 0.20 (0.06, 0.69) 0.011 | 0.15 (0.04, 0.55) 0.004 | 0.04 (0.01, 0.23) <0.001 |

Model 1: adjusted for age and gender.

Model 2: adjusted for age, gender, current smoking, diabetes mellitus, history of stroke, hypertension and heart rate.

Model 3: adjusted for age, gender, current smoking, diabetes mellitus, history of stroke, heart rate, hypertension, albumin, BUN, total cholesterol, total triglyceride, HDL-C, LDL-C, fasting glucose, serum creatinine, uric acid and serum phosphate.

*Odds ratios were calculated per 1-SD increment of serum calcium concentration (mmol/l).
